# Artificial sweetener is a growing threat for metabolic syndrome: why is extra attention required?

**DOI:** 10.2144/fsoa-2023-0092

**Published:** 2023-06-23

**Authors:** Sarvesh Sabarathinam, Dhivya Dhanasekaran, Nila Ganamurali

**Affiliations:** 1 Drug testing Laboratory, Interdisciplinary Institute of Indian System of Medicine (IIISM), SRM Institute of Science & Technology, Kattankulathur, Chennai, Tamil Nadu, 603203, India; 2 Clinical Trial Unit, Metabolic Ward, Interdisciplinary Institute of Indian System of Medicine (IIISM), SRM Institute of Science & Technology, Kattankulathur, Chennai, Tamil Nadu, 603203, India; 3 Certificate Program-Analytical Techniques in Herbal Drug Industry, Interdisciplinary Institute of Indian System of Medicine (IIISM), SRM Institute of Science & Technology, Kattankulathur, Chennai, Tamil Nadu, 603203, India

**Keywords:** bioanalysis, biochemistry, bioinformatics, cardiovascular, clinical pharmacology, endocrinology, epidemiology, metabolism, pharmacology, toxicology

“Metabolic syndrome (MetS) is a multifactorial cardiometabolic disorder that includes a cluster of complications, including hyperlipemia, elevated waist circumference and hypertension. MetS is not an independent entity known to be caused by a single element but are generally associated with cardiovascular risk factors and insulin resistance. As per WHO, EGIR, ATPIII (2001) and AAC3 (2003) guidelines, elevated blood glucose, obesity, abnormal waist circumference and elevated blood pressure are common contributing factors”. Patients identified with metabolic disorders are also more likely to develop Type 2 diabetes. The increased fatty tissue releases proinflammatory cytokines that affect insulin processing. Additionally, adipose tissue accumulation and tissue malfunction can result in insulin resistance [1]. Obesity provokes a state of chronic low-grade inflammation, leading to the disruption of insulin signaling pathways and further compromising insulin sensitivity [2].

Adults in the USA over 18 years of age continue to have a considerable prevalence of MetS. According to the previous literature survey, the occurrence of MetS increased by 35% between the 1980s and 2012 in the USA. The incidence was reported to be 25.3% in the 1980s and increased to 34.2% in 2012 [3]. The severity rate of MetS differs from one individual to another and has a more significant difference between race and ethnicity [4]. The prevalence of MetS exhibits global variation. In the USA, data from the National Health and Nutrition Examination Survey (NHANES) conducted between 2011 and 2016 revealed an estimated prevalence of approximately 34% among adult populations [5]. The Asia–Pacific region is experiencing a significant epidemic of MetS. Prevalence rates vary between countries, with approximately 1/5th or more of the adult population affected in most countries.

Dietary habits are directly related to symptoms of the MetS. Uncontrolled eating habits and sedentary lifestyles significantly worsen weight and cardiometabolic problems. Drastic change in the regular lifestyle such as diet, lack of physical exercise, alcohol consumption and disturbed sleep are the leading cause of metabolic disorder. Over 10 years, high consumption of junk foods in India increased the prevalence of overweight among school-aged children from 9.7 to 13.9% [6]. The first-choice treatment for patients with MetS aim to assist in the reduction of visceral fat, improve the sensibility to insulin and reduce the plasmatic concentrations of glucose and triglycerides and consequently decrease the risk factors which contribute to the development of Type 2 diabetes. This includes interventions such as a Mediterranean diet, statins, insulin therapy, nutraceuticals, butyrate andprobiotics, etc. [3].

## Artificial sweeteners

Artificial sweeteners are food additives that replicate the impact and flavor of sugar on taste, as the sensory characteristics of food are heavily influenced by taste, smell, texture and appearance. These are low-calorie sugar replacements that are utilized as a dietary choice in people with obesity or diabetes. These sweeteners are chemically synthesized or extracted from natural sources, and they are subjected to intensive testing for safety and regulatory approval before used in food items. Aspartame, sucralose, saccharin and stevia are all common artificial sweeteners [7].

### Widely used artificial sweeteners

- Aspartame is 180–200-times sweeter than sucrose. Aspartame may not meet expectations of being a healthier substitute for sugar in sweetened beverages because it has also been linked to Type 2 diabetes mellitus. The conversion of methanol by alcohol dehydrogenase during aspartame breakdown produces formaldehyde. Because aspartame is an exogenous source of formaldehyde, which can crosslink proteins with DNA, it may cause genotoxicity when consumed. Beyond metabolic disorders, aspartame can result in neuropsychiatric side effects such as depression, seizures and headaches. According to studies, aspartame and its metabolites raise the risk of neurodegenerative conditions such as brain tumors, Parkinson’s disease, multiple sclerosis and Alzheimer’s disease [8].
- Acesulfame K is one of the most common artificial sweeteners with little calories in the diet patterns followed to substitute high calories sugar is acesulfame-K (Ace-K). Ace-K is genotoxic and can prevent gut bacteria from fermenting glucose. One study demonstrated that acesulfame K increases the weight of male mice and alter the gut microbiome. Ace-K reveals gender-specific alterations in functional genes involved in energy metabolism in mouse models. Ace-K increased the body weight in male and had no effect on the body weight of female mice [9].
- Compared with aspartame, advantame is 116-times sweeter and sucrose is 37,000-times sweeter [10]. Severe intestinal problems were absorbed in high doses aspartame treated rabbits [10]. Despite this, bacterial assay reverse mutation test, it was discovered that advantame was not genotoxic [11].
- Erythritol has free radical scavenging activity, antioxidative and endothelium-protective properties and increases the malabsorption of fructose [12]. Ingestion of erythritol in healthy volunteers(>2 d) led to marked and prolonged increases in plasma erythritol levels which is directly proportional to increased platelet reactivity and thrombosis potential. These results show that erythritol increases the incidence of thrombosis and incidence of major adverse cardiovascular events includes death or nonfatal myocardial infarction or stroke [13].
- Maltotriose, is the second most abundant sugar in the world, and is utilized only in the later stages of alcoholic fermentation. The unsuccessful fermentation process in beer results in sedimentation of maltotriose which causes palatable and changes in ethanol concentration [14]. The biological activity of the phenolic chemicals in beer can be associated with cardiovascular risk and glucose metabolism when consumed moderately. On the other hand, excessive beer drinking may lead to weight gain and related morbidities [15].
- Neotame is an artificial sweetener that has received US FDA approval and is 7000–13,000-times sweeter than sugar. However, neotame safety investigations discovered that long-term neotame use is related to low body weight and weight gain, even though this has long been thought to be the result of insufficient food intake [16].
- More than 30 distinct steviol glycosides, including stevioside and rebaudioside-A, are found in *Stevia rebaudiana* leaves. Rebaudioside-A increases insulin production. However, the allergenic potential of stevia preparations was demonstrated in one investigation on the animal model. It is probably due to 95% steviol glycosides with sweetening properties [17].
- Raffinose is considered an anti nutritional factor since they cause flatulence in humans and animals [18].
- Saccharin is 300-times sweeter than sucrose. Reports show that the body cannot metabolise saccharin, unlike glucose and sucrose. Under various circumstances, saccharin has been reported as a stable chemical, and neither animal nor human studies have revealed any evidence of saccharin metabolism. Most papers mention that rats fed with significant dosages of saccharin develop bladder cancer more frequently. In rats, saccharin ingestion has been linked to adverse effects on most biochemical and haematological parameters. In diabetes and obese mice, consuming significant doses of saccharin (135 mg) may cause hypoglycemia, reduced hyperinsulinemia and lowered insulin resistance [19].
- Sucralose is an artificial organochlorine sweetener which is 600-times sweeter than sucrose. Sucralose changes the microbial composition of the GI tract in rats, with a disproportionately more significant loss of good bacteria. When sucralose cooks at high temperatures, hazardous chloropropanols are produced in large concentrations [20].

## Conclusion

While preserved foods and processed foods play a crucial role in our daily lives and are widely used in dietary and medicinal products, it is important to note that the extensive utilization of artificial sweeteners in various food items raises concerns regarding their safety. The evidence supporting these claims remains inconclusive, and significant toxicity and adverse effects have been observed in animal models. There needs to be more adequately designed randomized controlled studies to assess their efficacy in different populations. According to current evidence-based dietary recommendations, the use of artificial sweeteners, in general, is still debatable and consumers should be well-informed about their potential hazards.
